# Investigating the encapsulation of lead bromide perovskite with poly(3-bromothiophene) for improved aqua stability and enhanced fluorescence memory

**DOI:** 10.1098/rsos.241067

**Published:** 2025-02-05

**Authors:** Debasis Brahma, Jit Satra, Sayan Basak, Subhadeep Chakraborty, Rahul Chatterjee, Suman Acharya, Debdipta Basu, Abhijit Bandyopadhyay

**Affiliations:** ^1^ Department of Polymer Science and Technology, University of Calcutta, 92 A.P.C Road, Kolkata 700009, India; ^2^ Indian Institute of Engineering Science and Technology, Botanical Garden Area, Howrah, West Bengal 711103, India; ^3^ Indian Rubber Materials Research Institute, East Centre, Rubber Park, PO- Dhulagarh, P.S-Sankrail, Howrah, West Bengal 711302, India

**Keywords:** perovskite, FAPbBr_3_, encapsulation, stability enhancement, poly(3-bromo thiophene), fluorescence

## Abstract

Formamidinium lead bromide (FAPbBr_₃_) perovskites are promising candidates for optoelectronic applications owing to their exceptional semiconducting and photoluminescent properties. However, their high sensitivity to environmental factors like moisture and polar solvents limits their long-term stability, posing a barrier to commercial applications. This study addresses this stability challenge by encapsulating FAPbBr_₃_ in poly(3-bromothiophene) (PTBr), a high molecular-weight-conducting polymer, to enhance resistance to aqueous and solvent-based degradation. The PTBr encapsulation was found to significantly improve the thermal and environmental stability of FAPbBr_₃_, as evidenced by thermogravimetric analysis, which revealed a reduced and delayed mass loss and an increased residual mass (up to 28.17% in composites with 70% PTBr content). Photoluminescence studies demonstrated that the encapsulated composites exhibited a mean fluorescence lifetime of 87.4 ns, compared with 12.56% fluorescence retention in unencapsulated FAPbBr_₃_ after exposure to moisture for 45 days. Moreover, encapsulated FAPbBr_₃_ retained over 80% of its green light fluorescence intensity even after 1 year, whereas the unencapsulated sample degraded to less than 5%. Notably, the composites displayed fluorescence recovery upon exposure to polar solvents, further highlighting PTBr’s protective role. These findings provide a practical, non-interacting encapsulation strategy that enhances both the environmental and thermal stability of FAPbBr_₃_ while preserving its emission characteristics, offering potential to support the further development of perovskite-based optoelectronic devices for practical applications.

## 1. Introduction

Lead halide perovskites, particularly formamidinium lead bromide (FAPbBr_3_) nanocrystals, have garnered significant attention in the field of optoelectronics owing to their outstanding optical and electronic properties, such as high photoluminescence efficiency and tunable band gap [1–5]. These nanocrystals hold immense promise for applications ranging from solar cells to light-emitting devices [6,7]. However, a major impediment to their widespread adoption is their inherent instability, especially in the presence of moisture and heat, which can lead to degradation and performance loss in a quicker time [8–11]. Exposure to oxygen and moisture remains a primary obstacle to the commercial viability of metal halide perovskites, as these elements accelerate degradation. Additionally, thermal treatments cause morphological changes, while water molecules infiltrate the structure, forming hydrogen bonds with organic components and further reducing lifespan [12].

This study aims to develop strategies to enhance the stability and longevity of FAPbBr_₃_ nanocrystals for practical applications. Encapsulation offers a promising solution by creating a protective barrier around the perovskite crystals, shielding them from environmental degradation. Researchers have explored encapsulating perovskite crystals with organic polymers, using techniques like swelling–deswelling microencapsulation for effective dispersion and passivation. Composite films produced this way have shown high photoluminescence quantum yields (PLQY) of up to 48%, narrow colour purity (full-width half maximum (FWHM) of 18 nm), and extended fluorescence lifetimes (502 ns; [11]). Additionally, composites like MAPbBr_₃_-polystyrene and MAPbBr_₃_-polycarbonate have demonstrated the ability to withstand boiling water treatment for 30 min, with PLQY recovery rates of 15% and 7%, respectively [11–13].

Recent advancements in perovskite materials have focused on improving stability and efficiency. In 2020, Sardar *et al*. synthesized MAPbBr_₃_ nanocrystals within poly(3-bromothiophene) (PTBr), achieving bright green fluorescence and strong water resistance [14]. In 2016, FAPbBr_₃_-based solar cells with a lithium-modified TiO_₂_ interface showed high photovoltage (1.53 V) and over 8% efficiency [15]. More recently, in 2022, efforts to improve stability involved using polypyrrole and polymer encapsulants like poly(methyl methacrylate) and polycarbonate to protect CH_₃_NH_₃_PbI_₃_-_x_Cl_x_ films at temperatures as high as 1000°C [16].

Despite progress in developing stable perovskite nanocrystals, challenges remain. Encapsulation techniques for protecting perovskite from environmental degradation remain complex [17–19]. Maintaining consistent photoluminescence and recovery of fluorescence memory over time are key issues, along with the cost, scalability and compatibility of encapsulation methods with device integration. Moreover, ensuring that encapsulation does not compromise the performance of optoelectronic devices is critical. Fluorescence memory recovery is important, as it involves maintaining emission characteristics post-disintegration through re-crystallization [14–16]. Since 2017, significant advances have been made in improving the stability of metal halide perovskite composites, including caesium lead bromide (CsPbBr_₃_) in super-hydrophobic frameworks showing vibrant emission even after six months in water [20] and CsPbBr_₃_ composites with over a month of air stability [21]. Other composites, such as Methylammonium Lead Tribromide (MAPbBr_₃_)@SiO_₂_, demonstrated thermal stability for up to 60 h at 85°C [22], and CsPbBr_₃_/TiO_₂_ core/shell nanocrystals showed 12 weeks of water stability. Recent studies also highlight advances such as 1000 h humid air stability for formamidinium lead iodide (FAPbBI_₃_) films and poly(l-lactic acid) (PLLA)–FAPbBr_₃_ membranes retaining stability in water for 45 days [23–25].

Our study focuses on enhancing the long-term stability of FAPbBr_₃_ perovskites, which are highly susceptible to environmental degradation. Current encapsulation methods have shown limited success in addressing both moisture ingress and thermal stability under real-world conditions. We hypothesize that embedding FAPbBr_₃_ nanocrystals within a PTBr matrix will significantly improve stability, as PTBr’s bromine substituents provide superior hydrophobicity and environmental resistance [26]. PTBr is ideal for this purpose owing to its moisture resistance, high conductivity and good thermal stability, which are all crucial for improving optoelectronic device performance. By forming a robust encapsulation layer, PTBr prevents moisture ingress and enhances charge transport, offering advantages over traditional materials. Additionally, PTBr’s versatile polymerization allows for property customization, enabling optimization for various applications.

This study has two objectives: first, to synthesize FAPbBr_₃_ nanocrystals with controlled morphology, and second, to encapsulate them in PTBr matrices, creating composites with improved environmental stability and fluorescence memory. Our approach aims to overcome existing limitations in stability for both inorganic and hybrid perovskites, with PTBr encapsulation providing better resistance without affecting emission behaviour. We will systematically characterize these composites to validate our hypothesis and explore how polymeric encapsulation preserves the optical and structural properties of lead halide perovskites under challenging conditions, contributing insights into real-world applications.

## 2. Experimental procedure

### 2.1. Materials

Both lead carbonate basic (PbCO_3_, Pb(OH)_2_ 75–80%) and hydrobromic acid (HBr, 47%) were purchased from Merck India Ltd. Cetyltrimethylammonium bromide (CTAB, 99%) was procured from Loba Chemie, and 3-bromothiophene (C_4_H_3_BrS, 98%) and N,N-dimethylformamide (DMF) were obtained from Sigma Aldrich, USA. Toluene and chloroform of Analytical Reagent (AR) grade were obtained from indigenous sources.

### 2.2. Synthesis of bulk formamidinium lead bromide

In a 25 ml conical flask, 2.5 g of basic lead carbonate was combined with 2.4 ml of HBr. The mixture was stirred at room temperature until no bubbles had appeared. In another flask, 2 ml (0.036 mol) of HBr and 1.04 g (0.01 mol) of formamidine acetate were carefully mixed. The lead carbonate solution was then added to this mixture and stirred at room temperature for an additional 30 min. Afterwards, the excess acid was washed away with acetone, and the mixture was filtered and then dried in a vacuum oven to yield an orange solid.

### 2.3. Synthesis of formamidinium lead bromide nanocrystals

In a 100 ml conical flask, 0.04 g of previously synthesized FAPbBr_3_ and 0.03 g of CTAB were added. Then, 2 ml of DMF was added to the flask, and the mixture was agitated for approximately 25 min at 25°C. Finally, 50 ml of toluene was gradually added to the mixture to facilitate the formation of orange perovskite nanocrystals at the same temperature. The resultant product was then separated by centrifugation, and the orange solid was dried for 48 h at room temperature in a vacuum oven.

### 2.4. Synthesis of poly(3-bromothiophene)

To a 100 ml conical flask, 7 g of anhydrous ferric chloride and 15 ml of chloroform were added under a nitrogen atmosphere with vigorous stirring. The solution was then cooled to a temperature between 0°C and 4°C using an ice bath. Next, 1.75 g of 3-bromothiophene dissolved in 10 ml of chloroform was added dropwise while stirring. Once the addition was complete, the mixture was stirred for an additional 6 h, maintaining the temperature between 0°C and 4°C. Afterwards, it was stirred at room temperature (25°C) for another 24 h. Following this, the resultant product was filtered, washed with water and ethanol and finally dried in a vacuum oven, yielding a brown-coloured product.

### 2.5. Synthesis of formamidinium lead bromide perovskite–poly(3-bromothiophene) composites via *in situ* technique

In this process, the FAPbBr_3_ nanocrystals were synthesized in the presence of PTBr for an impromptu encapsulation. Into a 100 ml conical flask, 0.04 g of previously synthesized FAPbBr_3_ and 0.03 g of CTAB were added in the presence of 0.02 g PTBr. To it, 2 ml of DMF was added, and the mixture was agitated for approximately 25 min at 25°C. After that, 50 ml of toluene was gradually added to the mixture to promote the formation of perovskite nanocrystals at the same temperature. The resulting product was separated by centrifugation and then dried for 48 h at room temperature in a vacuum oven. The amount (mass) of the PTBr initially added to the reaction mass was varied to investigate the extent of encapsulation in the nanocrystals within the resultant composites designated as Pv/PTBr(1)–Pv/PTBr(6). The four compositions with details are stated in table 1.

**Table 1.** Sample composition with relevant properties of Pv, PTBr and Pv/PTBr-based composites. (PV, perovskite; PTBr, poly (3-bromo thiophene); TGA, thermogravimetric analysis; FAPbBr_3_, formamidinium lead bromide.)

sample designation	Pv (%)	PTBr (%)	residue after TGA	band gap (eV)	average lifetime (ns)
**FAPbBr_3_(Pv**)	100%	0%	0%	3.49	7.23
**PTBr**	0%	100%	23.98%	3.13	421
**Pv/PTBr(1**)	80%	20%	5.21%	3.47	85.4
**Pv/PTBr(2**)	50%	50%	9.23%	3.53	87.4
**Pv/PTBr(4**)	45%	55%	8.5%	3.53	38.5
**Pv/PTBr(6**)	30%	70%	28.17%	3.46	14.3

## 3. Characterization

Gel permeation chromatography was conducted to determine the molecular weight and molecular weight distribution of PTBr. It was conducted using a spectroscopic grade model and DMF as a solvent. Thermogravimetric analysis (TGA) of the pure and composite samples was carried out using a TGA Q-50 instrument procured from TA Instruments to investigate the thermal and oxidative stabilities and residue preparation for quantitative estimation of the encapsulant. The scans were conducted under both inert (N_2_) and oxidative (O_2_) atmospheres, spanning from ambient temperature 20°C to 700°C at a heating rate of 20°C min^−1^, maintaining a constant mass flow rate of 50 ml min^−1^.

Fourier-transform infrared (FTIR) spectra of the samples were obtained using a Perkin Elmer FTIR spectrum two spectrophotometer, covering the spectral range of 400−4000 cm^−1^ with a peak resolution of 4 cm^−1^. The measurements were conducted in attenuated total reflectance mode using a diamond crystal to find out the chemical structure and the nature of interaction prevailing between FAPbBr_3_ and PTBr. The ultraviolet-visible (UV-Vis) absorption spectra were recorded in the JASCO V-530 spectrophotometer. Photoluminescence (PL) spectra and time-correlated single photon counting lifetime measurements were performed using a Horiba PL measurement system equipped with a 330 nm diode. Both studies were conducted to determine the optoelectronic behaviour of the samples. Both encapsulated and unencapsulated FAPbBr_3_ were green emitters. The intensity of the green light during the stability analysis and fluorescence memory recovery analysis was quantified using MATLAB software following a similar method developed by our research group as reported previously [27]. This approach enabled the faster and accurate quantification of the emitted green light relative to the total light emitted by the samples, providing a clear assessment of the fluorescence properties under investigation.

## 4. Results and discussion

### 4.1. Synthesis of formamidinium lead bromide nanocrystals and composite with poly(3-bromothiophene)

**Figure 1.**
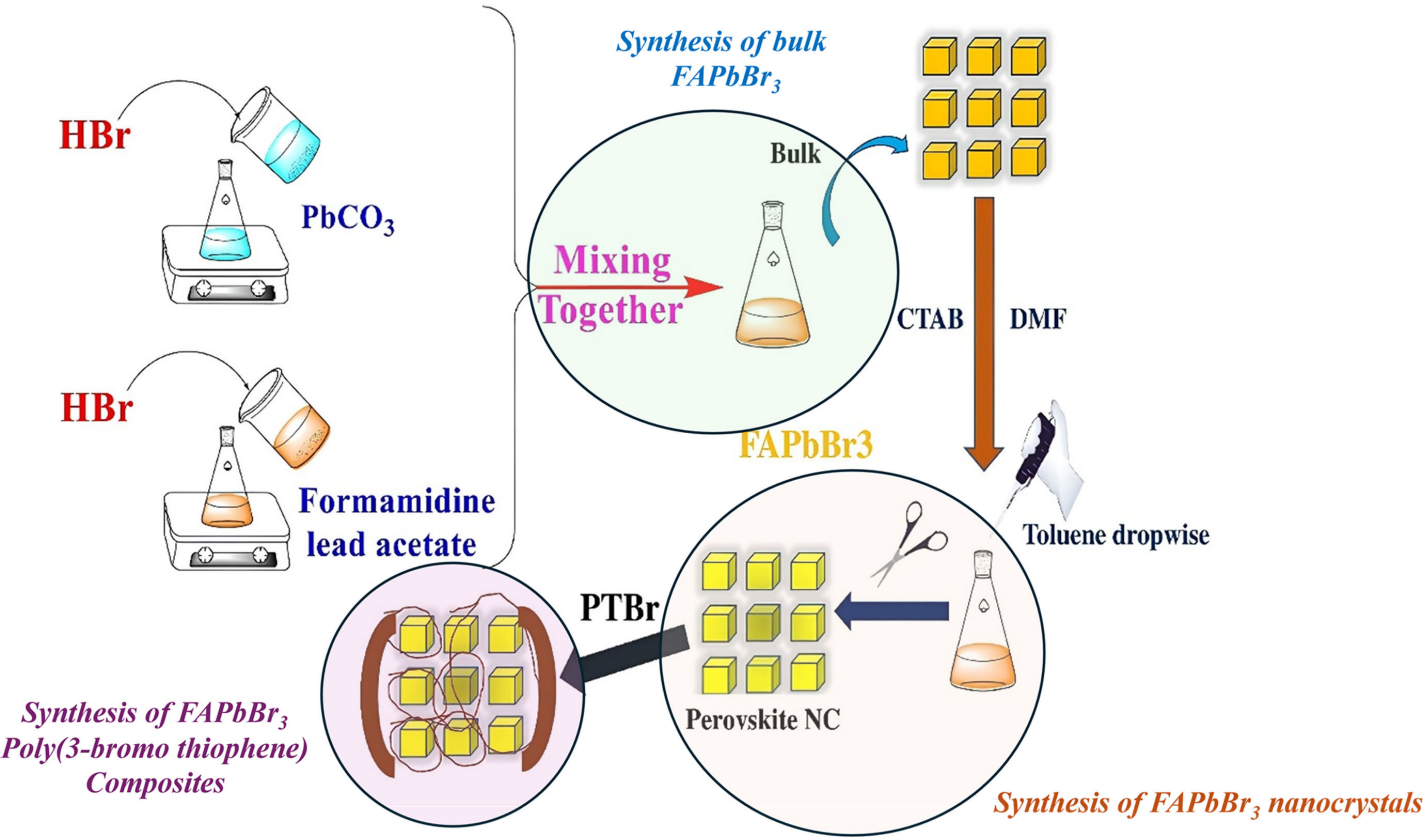
Illustration showing the synthesis of FAPbBr_3_(Pv) nanocrystals and encapsulation with PTBr.

The synthesis route to FAPbBr_3_ nanocrystals and its composites with PTBr is schematically shown in figure 1. Initially, HBr reacted with both PbCO_3_ and formamidine lead acetate and produced the bulk FAPbBr_3_ salts, having an octahedral structure. Subsequent treatment with CTAB, a tertiary amine, facilitated the formation of nanocrystals through adsorption and segregation of the bulk octahedrons. Once that was optimized, the same stoichiometry and experimental conditions were used to re-synthesize the Pv nanocrystals in the presence of PTBr. Bulk Pv was added along with CTAB, DMF and finally toluene for synthesis, impromptu encapsulation and purification of the nanocrystals. Such a method was adopted to exploit the diffusion, passivation and hydrophobicity of Br^−^, to improve the stability and fluorescence memory.

Furthermore, *in situ* soaking of hydrophobic and thermally stable PTBr proved to be an effective method for enhancing long-term stability against humidity and environmental factors. The synthesized PTBr exhibited high molecular weights, with a number average molecular weight (*M*_n_) of 1 684 908 Da and a weight average molecular weight (*M*_w_) of 1 901 245 Da, resulting in a narrow polydispersity index (PDI) of 1.12 (electronic supplementary material, figure S1). The molecular weight of PTBr was selected to balance its solubility, processing properties and encapsulation efficiency. PTBr, with the molecular weight chosen, allows for effective encapsulation of FAPbBr_₃_ nanocrystals without excessively increasing the viscosity of the solution. This ensures a stable dispersion of the nanocrystals during the synthesis process, facilitating uniform encapsulation. Additionally, the chosen molecular weight helps to maintain the desirable mechanical and thermal properties of the composite, which are crucial for enhancing the stability and durability of the perovskite nanocrystals under environmental stress.

These eventually would enhance the stability and resist degradation of the optoelectronic devices [28,29]. Furthermore, the presence of the ammonium group in the cetyl trimethyl ammonium cation (CTA^+^) has proved to be effective in diminishing trap states on the surface of the Pv materials [23]. This reduction not only leads to an enhancement in device efficiency but also plays a crucial role in bolstering stability as well [23].

Moreover, modifying the doping concentration of Br^−^ has been found to positively influence energy levels and crystal grain growth, offering a straightforward means to reinforce the structural integrity and improve the performance of perovskite crystals [28,29]. Additionally, the hydrophobicity provided by the long carbon chains of CTAB along with PTBr as already mentioned enhances the device’s resistance to moisture, further increasing its durability in various environmental conditions [28–30].

### 4.2. Thermal degradation analysis

To assess the thermal stability of the synthesized materials and to quantify the actual Pv–PTBr ratio in the derived composites, the TGA was conducted. The TGA thermograms are shown in the electronic supplementary material, figure S2. The Pv nanocrystals have exhibited two-stage degradation where the first stage recorded about 48.29% mass loss starting from 223°C under N_2_ and thereafter about 49.34% mass loss until 600°C under O_2_, respectively (electronic supplementary material, table S2).

The first mass loss was probably owing to the degradation of the organic components, while the second one was owing to the oxidative disintegration and simultaneous elimination of volatile bromide from the lead salt. The residue obtained was negligible, which was assumed to be nearly zero. On the contrary, PTBr exhibited an apparently single-stage degradation starting from 400°C under O_2_ and eventually ended up with a residue of 23.98% at 600°C. Notably, all the Pv/PTBr composites exhibited a hybrid mass loss characteristic to both the components. Pv/PTBr(1) displayed the first mass loss of 47.47% at around 223°C and a second mass loss of 45.85% at 420°C and led to a meagre residue of 5.21% (electronic supplementary material, table S1). The latter was assumed to be composed of the carbon residue obtained from the degraded mass of PTBr. Similarly, Pv–PTBr(2), Pv–PTBr(4) and Pv–PTBr(6) displayed the first and second mass losses of 45% at 244°C and 43.47% at 459°C; 33.31% at 246°C and 55% at 335°C and 19.60% at 289°C and 47.77% at 426°C, respectively (electronic supplementary material, table S1).

It is evident that the adsorption of PTBr has enhanced both oxidative and non-oxidative thermal stabilities in the form of reduced and delayed mass losses and the onset of degradation, particularly in the first stage as the proportion of PTBr was increased. However, the final residue amounts did not comply with the initial content of PTBr in the reaction mixture (electronic supplementary material, table S1). In the cases of Pv–PTBr(1) and Pv–PTBr(6), the PTBr amount was found to be slightly higher than the theoretical value, while in the other two cases it was slightly lower, elucidating the formation of higher and lower thicknesses of the encapsulants over the Pv nanocrystals, respectively (electronic supplementary material, figure S2).

**Figure 2.**
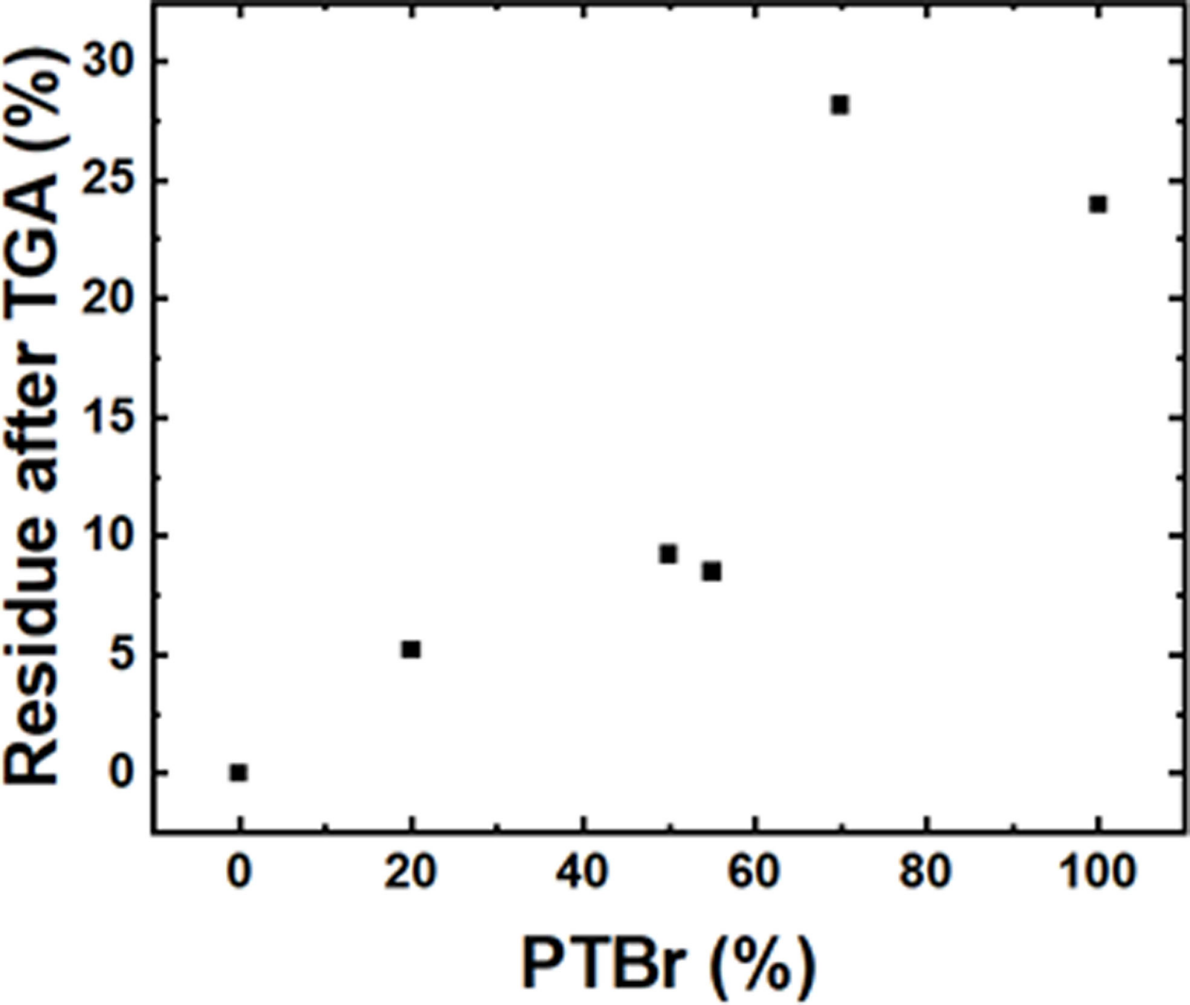
Effect of PTBr on the thermal stability of FAPbBr₃, expressed as the residue after thermogravimetric analysis (TGA).

The TGA results demonstrate the significant impact of polymer encapsulation on the thermal stability of FAPbBr_₃_. The data indicate that both the composition and the quantity of PTBr in the Pv/PTBr composites play a crucial role in enhancing the thermal stability of FAPbBr_3_. Notably, the composite designated as Pv/PTBr(6) (figure 2) shows exceptional effectiveness in improving resistance to thermal decomposition. This enhanced stability may be attributed to the specific interactions between PTBr and FAPbBr_₃_, as well as the barrier properties that PTBr provides against degradation products at elevated temperatures.

As the percentage of PTBr increases, a noticeable rise in the residue after TGA analysis is observed, highlighting the importance of PTBr in retaining material integrity under thermal stress. For instance, while the FAPbBr_₃_ (Pv) sample with 100% polymer content yielded no residue, the pure PTBr sample exhibited a residue of 23.98%. By contrast, composites with varying PTBr content, such as Pv/PTBr(1) with 20% PTBr, resulted in a residue of only 5.21%. However, the residue significantly increased to 28.17% for the Pv/PTBr(6) composite, which contains 70% PTBr (figure 2).

### 4.3. Fourier-transform infrared spectroscopic analysis

**Figure 3.**
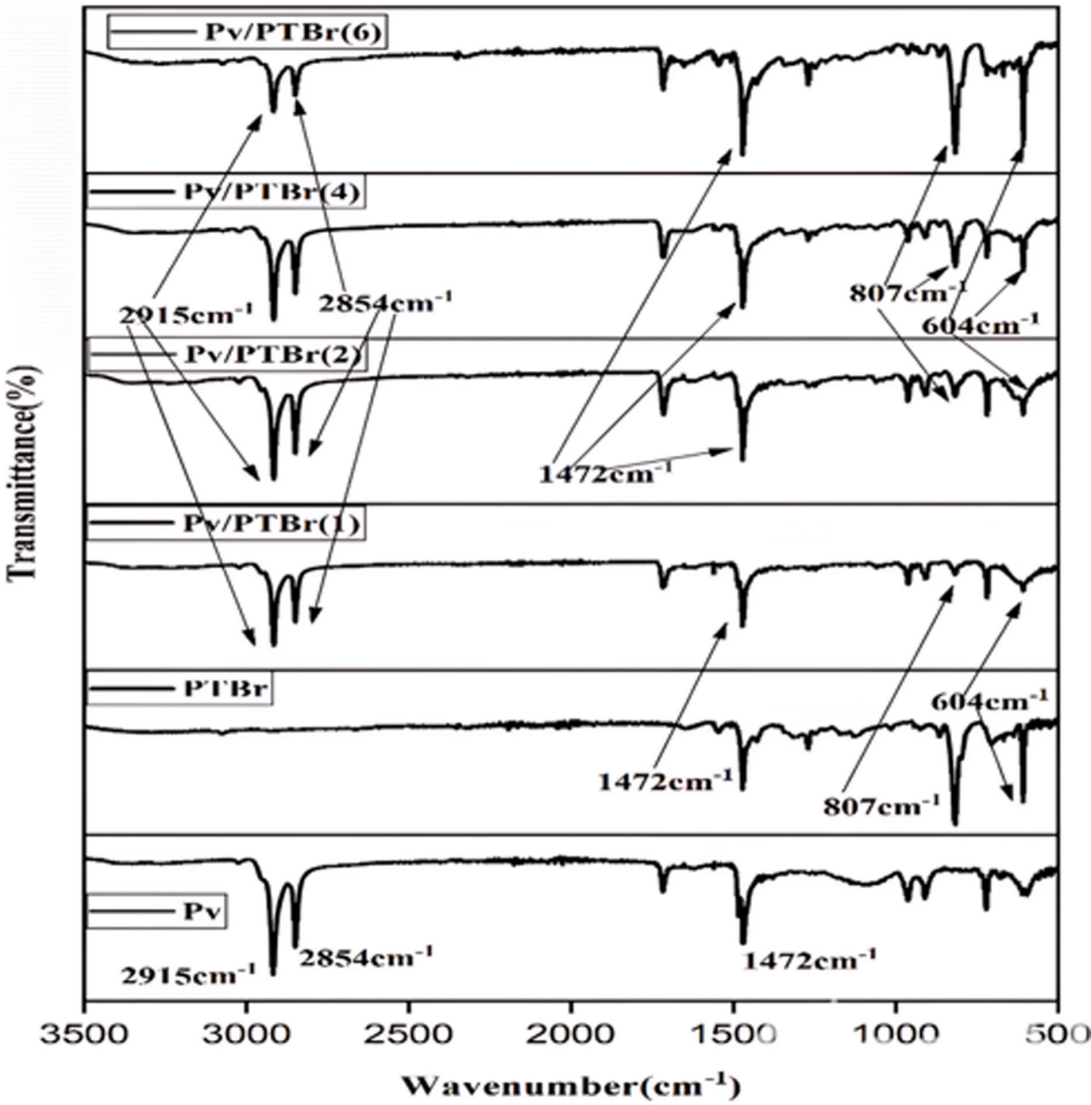
FTIR analysis of Pv, PTBr and Pv/PTBr-based composites.

FTIR spectra of FAPbBr_3_, PTBr and their composites are displayed in figure 3. The sharp peaks detected at 2915 and 2854 cm^−1^ corresponding to the C–H stretching vibrations of organic components were noted in both FAPbBr_3_ and the composites [31,32]. Additionally, a distinctive peak at 1472 cm^−1^, attributed to the C=C stretching vibrations, was also noted for FAPbBr_3_, PTBr and the composites (Pv/PTBr(1)–Pv/PTBr(6); [33]) as well. The presence of these peaks evidently confirms the deposition of PTBr over FAPbBr_3_ in the sample under study [34]. In addition to these, the specific bands observed at 807 and 604 cm^−1^, are attributed to C–S bending and the C–Br stretching vibrations, respectively, and in both PTBr and all the composites, further reinforced the adsorption phenomenon. The respective transmittance in the composites neither shifted from pure Pv nor from pure PTBr; hence it indicates the exitance of physical interaction between the nanocrystals and PTBr.

### 4.4. Ultraviolet-visible spectroscopic analysis

**Figure 4.**
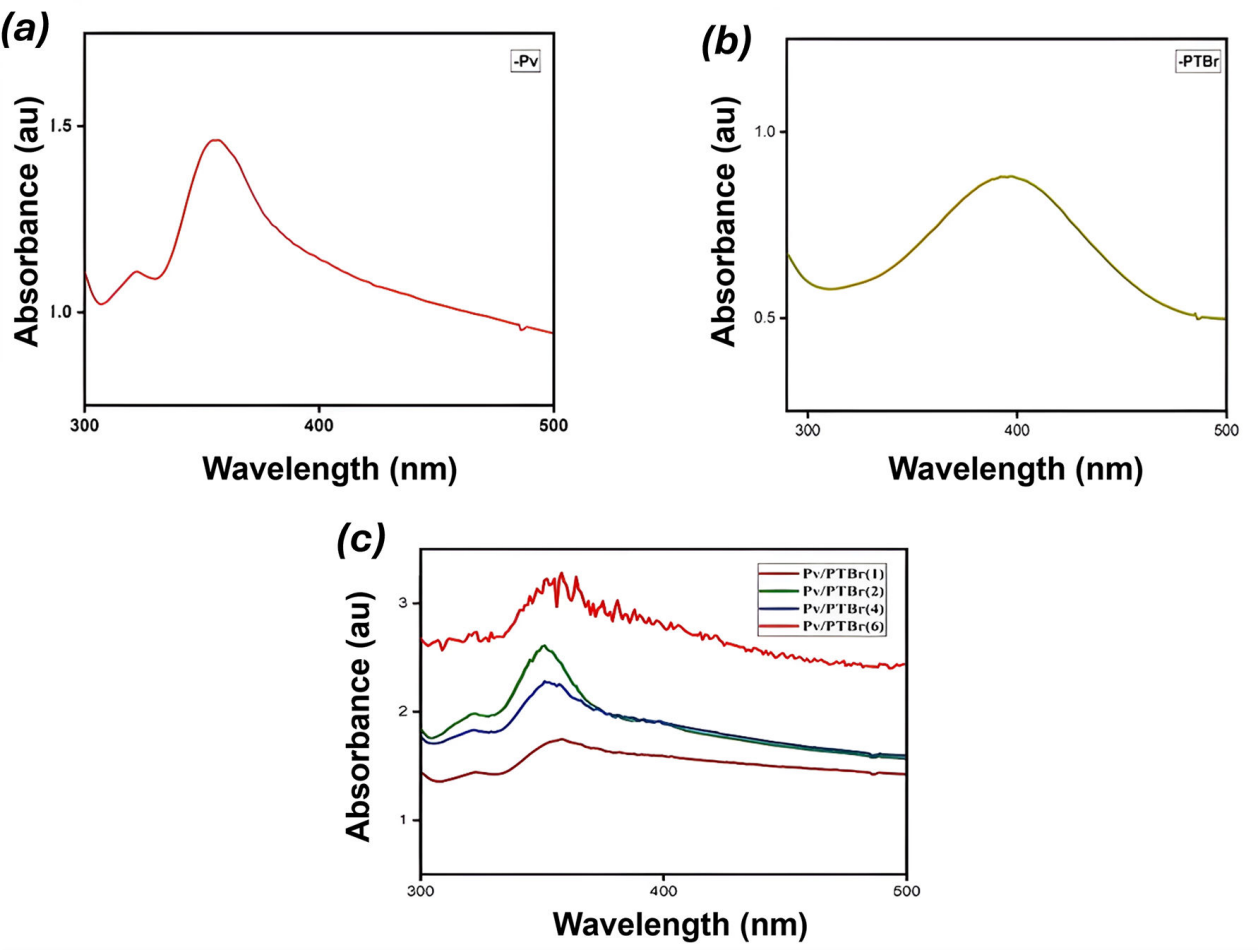
UV-Vis spectrum of (*a*) Pv, (*b*) PTBr and (*c*) Pv/PTBr-based composites.

The UV-Vis spectrum of unencapsulated FAPbBr_3_, PTBr and the Pv/PTBr(1–6) composites is displayed in figure 4. The FAPbBr_3_ nanocrystals exhibited an adsorption maximum at 355 nm because of electronic transition (figure 4*a*). The π–π^*^ transition in pure PTBr was obtained at 398 nm (figure 4*b*), which was weaker than the transition noted in FAPbBr_3_. All the composites had shown the electronic transition of FAPbBr_3_ as the primary transition. It was slightly shifted to 357 nm in Pv/PTBr(1), 351 nm in both Pv/PTBr(2) and Pv/PTBr(4) and 358 nm in PV/PTBr(6), respectively, as compared with the pure FAPbBr_3_.

Pv/PTBr(1) and Pv/PTBr(6) demonstrated enhanced absorbances compared with the other compositions, although a definitive explanation for the unique behaviour of Pv/PTBr(6) remains elusive [13,15,26]. Near retention of the absorption maxima of the Pv nanocrystal in the composites, along with no clear evidence of the absorption maximum of PTBr, establishes that PTBr did not chemically interact with FAPbBr_3_. This lack of chemical interaction consequently affects the band gap energies as reported in table 1. The data were calculated using the following empirical equation (4.1):


(4.1) $$E_{g}=1240/\lambda \left(in\;eV\right).$$

The band gap in pure FAPbBr_3_ nanocrystals was 3.49 eV; it was reduced to 3.13 eV in PTBr for which the absorption maxima in the latter had shown a blue shift. The composites, however, had shown a marginal deviation from FAPbBr_3_, e.g. Pv/PTBr(2) and Pv/PTBr(4) exhibit higher band gaps, while Pv/PTBr(1) and Pv/PTBr(6) exhibit the reverse (table 1) primarily owing to the absence of strong chemical interaction between FAPbBr_3_ and PTBr. Parallelly, it also implies that the conducting polymer could not affect the fundamental electronic structure of FAPbBr_3_ as well [34,35]. However, the slight deviation in band gap energy was probably owing to the variation in the extent of adsorption of PTBr at different stoichiometric compositions.

### 4.5. Analysis of emission spectra and time decay

**Figure 5.**
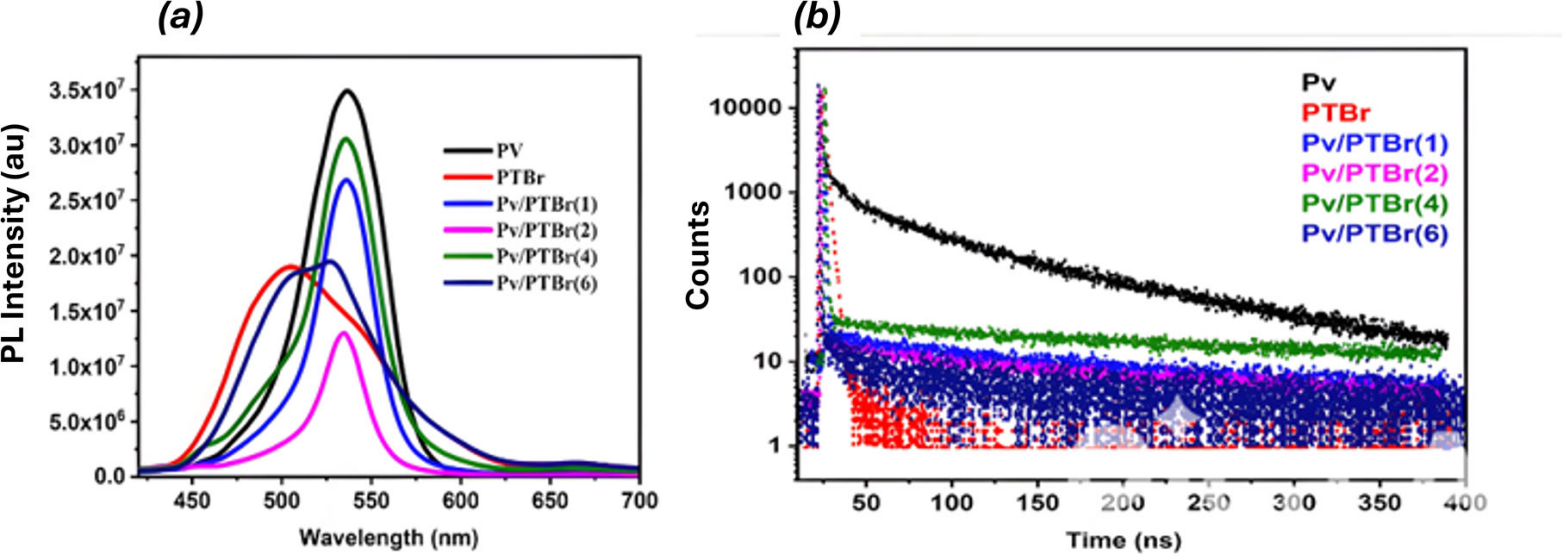
(*a*) PL spectrum of Pv, PTBr and Pv/PTBr composites. (*b*) Time-resolved photoluminescence of Pv, PTBr, Pv/PTBr composites.

**Figure 6.**
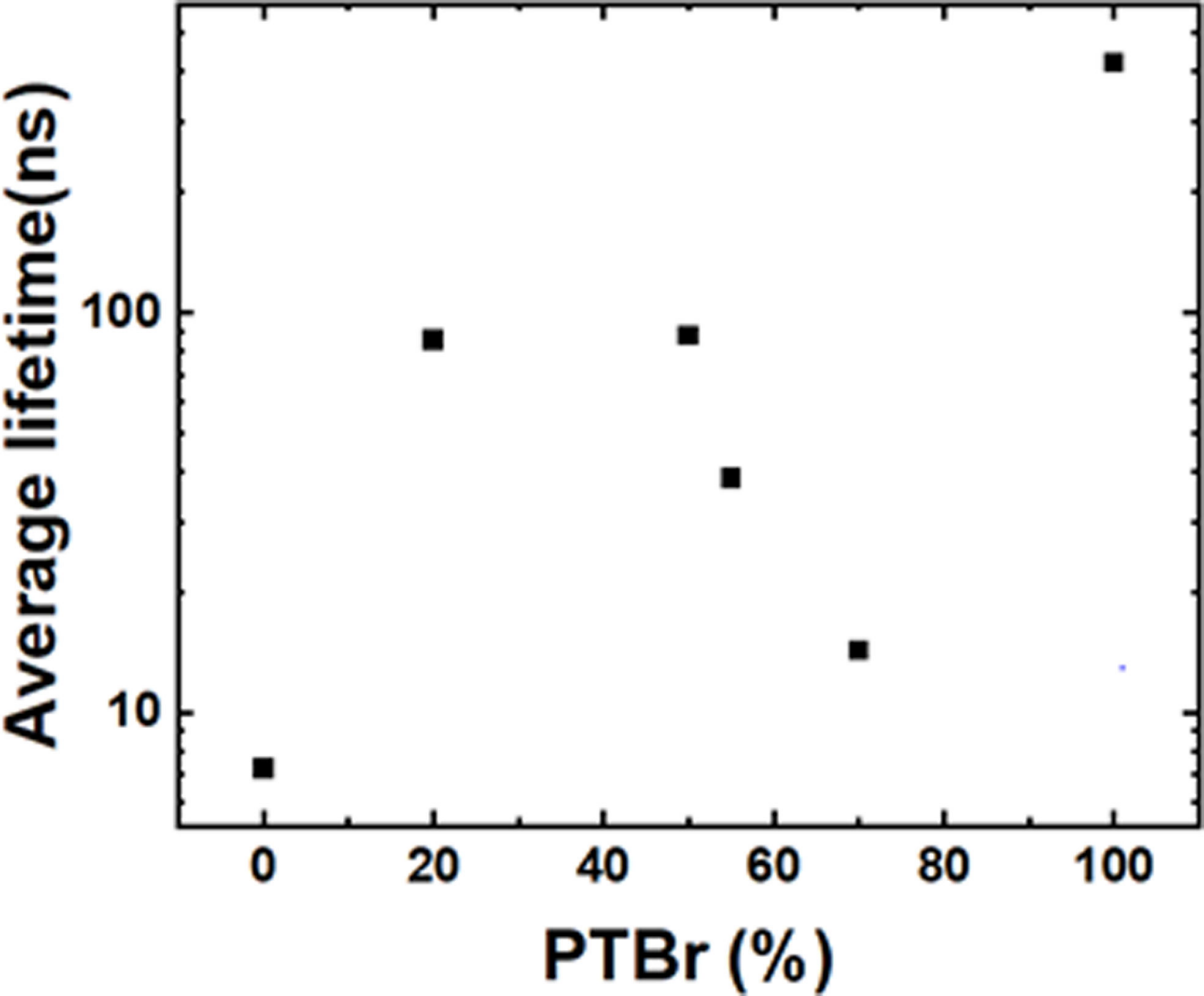
Average lifetime of PV, PTBr and PV/PTBr composites reported as increasing with PTBr concentration.

The PL emission spectra of pure Pv, PTBr and their composites are exhibited in figure 5*a*, the emission maximum of FAPbBr_3_ was obtained at 536 nm with an FWHM of 50.67 nm while that of neat PTBr was obtained at 509 nm with a lower emission intensity and broader FWHM of 87.20 nm. The latter suggests a characteristically different emission behaviour possessed by the conducting polymer from that of the inorganic crystal. The PL spectra of FAPbBr_3_/PTBr composites showed a gradual shift in emission maxima towards the emission maximum of pure PTBr as its stoichiometric composition was increased. The composites from Pv/PTBr(1) to Pv/PTBr(4) exhibited the emission behaviour resembling that of unencapsulated FAPbBr_3_, while Pv/PTBr(6) displayed the maximum close to that of the neat PTBr, respectively, as shown in figure 5*a* [35–38]. All the samples, however, had shown green fluorescence as observed from visual images in figure 6. Notably, the FWHM values of the emission spectra vary across the composites, with Pv/PTBr(2) exhibiting a narrower FWHM (26.14 nm) compared with the others. A narrower FWHM suggests stronger physical binding between PTBr and FAPbBr_3_, leading to excellent optical PL properties. However, it had shown a lower emission intensity compared with the other composites; it was probably owing to an enhanced photo-induced charge separation behaviour between the components. The time-resolved photoluminescence spectra of the pure and the composite samples are shown in figure 5*b*. The mean average lifetime (*T*_avg_) was calculated using equation (4.2):

(4.2) $$T_{avg}=\frac{\sum B_{i}T_{i}^{2}}{\sum B_{i}T_{i}},$$

where *B*_*i*_ represents the pre-exponential factor of the *i*th decay time constant (*T*_*i*_).

The spectra have revealed significant changes in the relaxation behaviour. Notably, all the Pv/PTBr composites exhibited delayed lifetime values than the unencapsulated FAPbBr_3_(Pv), with Pv/PTBr(2) showing the longest average lifetime (87.4 ns) among all composite samples (figure 6). The delayed relaxation as shown by all the composites was owing to improved charge separation between FAPbBr_3_ and PTBr through physical bonding as already mentioned. Parallelly, the most delayed relaxation as exhibited by Pv/PTBr(2) contemplated the highest FWHM and excellent surface passivation effect through the maximum extent of charge transfer between Pv and PTBr, respectively (figure 6).

### 4.6. Stability analysis of the pure and the encapsulated formamidinium lead bromide against moisture and fluorescence memory recovery

**Figure 7.**
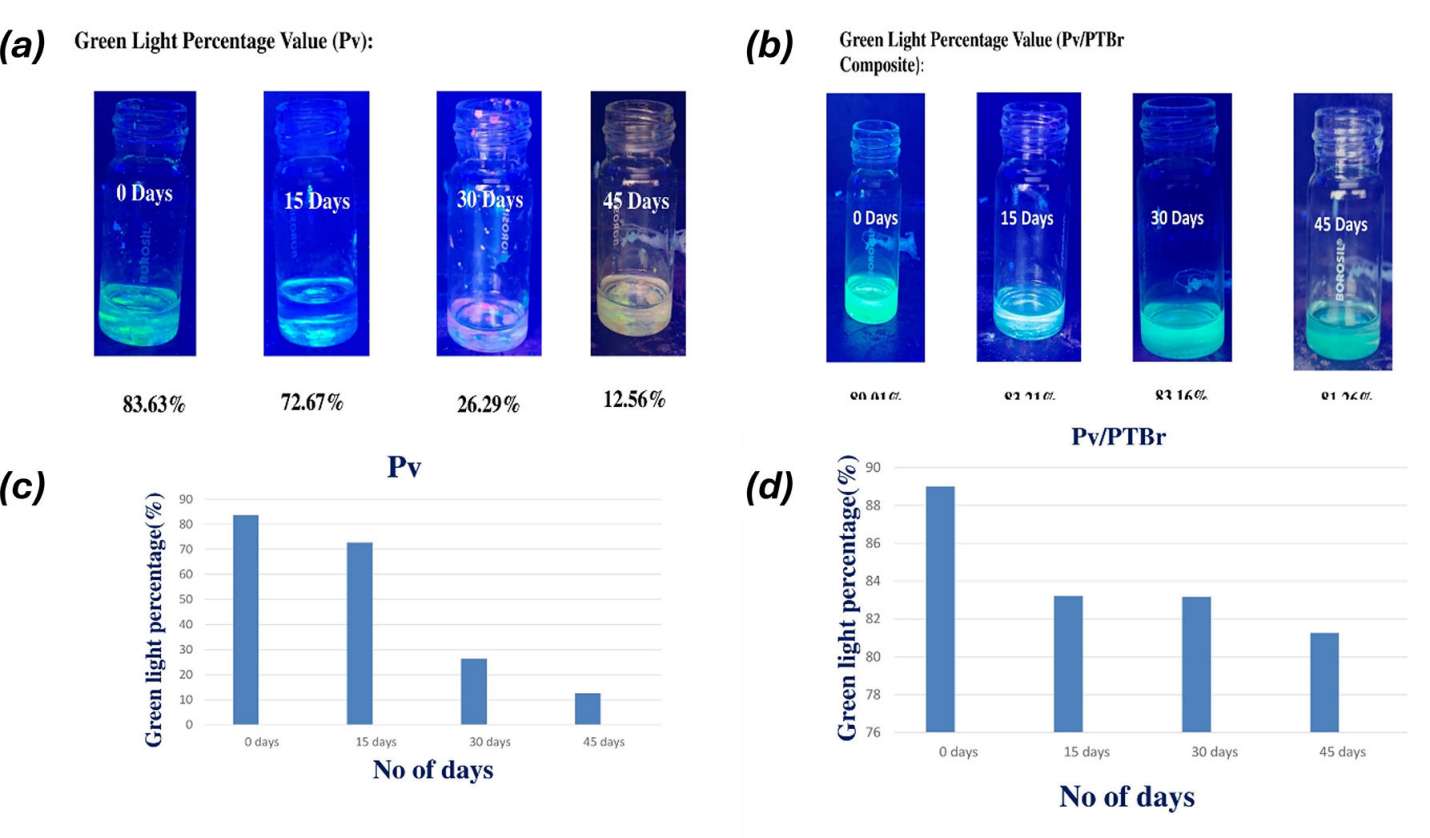
Long-term stability test of Pv/PTBr(2) stored in aqua moist conditions kept up to 0 days to 45 days of study. (*a*) Green light percentage value of Pv. (*b*) Green light percentage value of Pv/PTBr composite. (*c*) and (*d*) The plot of green light percentage versus number of days of Pv and Pv/PTBr composite.

**Figure 8.**
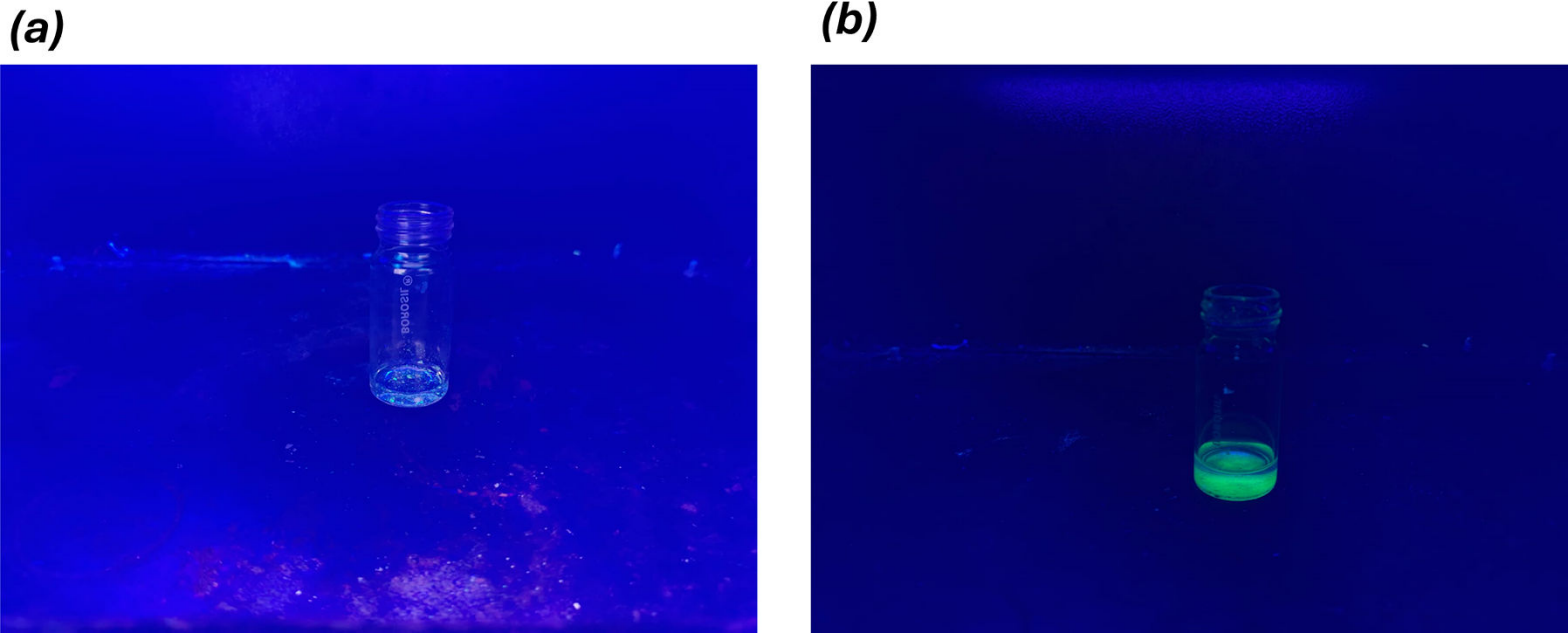
Luminescence intensity after 1 year: (*a*) PV is <5% and (*b*) PV/PTBr(2) is 87%.

Since Pv/PTBr(2) evolved as the best composite sample, it was further characterized for long-term stability and fluorescence memory recovery to unveil the application potential. During the experiments, both unencapsulated FAPbBr_3_ and encapsulated FAPbBr_3_(Pv/PTBr(2)) were exposed to prolonged (45 days) wet conditions, and their green light fluorescence was monitored in an ultraviolet chamber over time (figure 7). One may note a gradual decrease in the green light fluorescence of the unencapsulated Pv in the UV chamber occurred with the time of exposure, manifesting the proneness to degradation. Using MATLAB software, the emission intensities were quantified and mentioned below each figure. The unencapsulated FAPbBr_3_ gradually lost the fluorescence intensity from 83.63% to 12.56% during the exposure time, while for Pv/PTBr(2) the loss was only a meagre intensity from 89.01% to 81.26% during the same exposure period. Owing to the water-resistant encapsulation of PTBr over FAPbBr,_3_ the latter was greatly stabilized, while without any PTBr the FAPbBr_3_ nanocrystals disintegrated and transferred to macroform, reducing the fluorescence intensity. The extended long-term stability of the same encapsulated FAPbBr_3_ is illustrated through figure 8, which highlights the exceptional PL behaviour that was retained even after 1 year of exposure in complete contrast with the neat perovskite nanocrystals that exhibited water failure. The encapsulated sample maintains its green light fluorescence over the course of 1 year luminescence intensity of 80%, showcasing the effectiveness of the encapsulation strategy in preserving the optical properties of the perovskite crystal under challenging environmental conditions. By contrast, the control perovskite crystal, which lacked the protective encapsulation layer, experienced rapid degradation and significant loss of PL over time (with luminescence intensity dropping below 5%). This highlights the vulnerability of unencapsulated perovskite crystals to environmental factors such as moisture and chemical exposure.

Research on the stability of perovskite materials has yielded encouraging results across a range of compositions and composite structures. For example, PLLA–FAPbBr_₃_ nanofibrous membranes have been reported to maintain stability for 45 days. Additionally, films composed of FAPbBI_₃_/ammonium lead iodide (FAPbBI_₃_/A_₂_PbI_₄_) demonstrated extended stability of up to 1000 h. A noteworthy stability period was observed in CPB@SHFW composites, which remained stable for six months, whereas CsPbBr_3_–polypyrrole (CSPbBr_₃_–polypyrrole) composites retained their structural integrity and functional stability for 30 days.

Among these, FAPbBr₃–PTBr composites stood out owing to their extended stability of over 1 year, attributed to the protective PTBr layer that significantly improved their PL properties by acting as a barrier against degradation. Table 2 provides a comprehensive summary of recent findings on the stability of various perovskite materials, comparing their lifespans and enhancing factors [20–26].

**Table 2.** Recent reported work on enhancing the stability of different types of perovskite materials. (PTBr, poly(3-bromo thiophene); TGA, thermogravimetric analysis; FAPbBr_3_, formamidinium lead bromide; CPB, CsPbBr_3_ quantum dots; SHFW, super-hydrophobic porous organic polymer frameworks.)

materials	stability	uniqueness	reference
FAPbBr_3_–PTBr composites	more than 1 year	exceptional long-term stability; superior photoluminescence retention; robust encapsulation against environmental degradation	this work
CPB@SHFW	more than six months	long-term stability in water; high resistance to environmental factors	[20]
FAPbBr_3_–polypyrole	not mentioned	effective for short-term stability in certain environments; good for flexibility in device applications	[15]
CsPbBr_3_−PMMA, CsPbBr_3_−PBMA and CsPbBr_3_−PS composites	more than one month	various combinations for enhanced thermal stability and photoluminescence properties	[21]
MAPbBr_3_@SiO_2_ composites	60 h stability at 85°C and 350 h at 450 nm light	high stability at elevated temperatures and light exposure	[22]
CsPbBr_3_/TiO_2_ core/shell NCs	more than 12 weeks	promising water stability; effective core/shell structure enhances stability	[23]
FAPbBI_3_/A_2_PbI_4_ films	1000 h	high stability in controlled environments; significant longevity compared with earlier composites	[24]
PLLA-FAPbBr_3_ nanofibrous membranes	45 days	good stability for intermediate durations; suitable for flexible applications	[25]
CsPbBr_3_/polypyrrole (PPy)	30 days	strong stability in specific environmental conditions; potential for integration into flexible devices.	[26]

**Figure 9.**
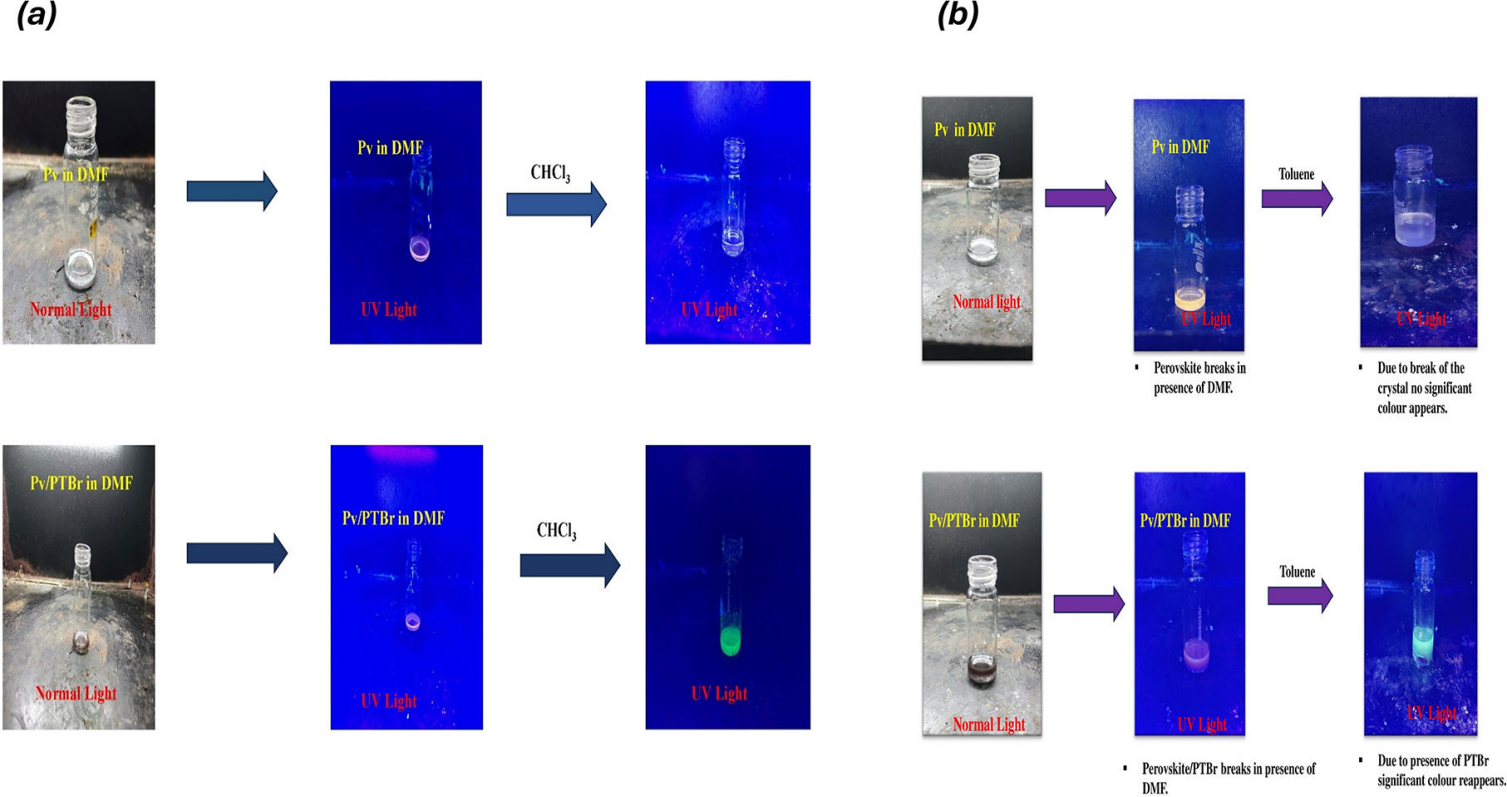
Fluorescence recovery test of PV and PV/PTBr composite: (*a*) CHCl₃, (*b*) toluene.

Further investigations involved a fluorescence recovery test, where both Pv/PTBr(2) and unencapsulated Pv were exposed to the polar solvent DMF. This exposure resulted in orange coloration, indicative of crystal breakdown in both the samples owing to the solvent’s penetrations within FAPbBr_3_ (figure 8). However, the subsequent addition of the non-polar solvent toluene enabled the encapsulated Pv to regain its green light fluorescence, highlighting the protective role of PTBr in maintaining fluorescence properties under adverse conditions. By contrast, the unencapsulated Pv did not exhibit fluorescence recovery, emphasizing the importance of encapsulation in preserving optical characteristics. The critical role of PTBr in facilitating fluorescence recovery was evident as DMF quickly dissolved PTBr molecules, leading to crystal disintegration and reduced fluorescence. Reintroducing toluene allowed PTBr to recombine over the elastic FAPbBr_3_ structure, stabilizing and reactivating fluorescence properties. Additionally, similar results were obtained when employing another non-polar solvent, CHCl_3_ (chloroform), in place of toluene, demonstrating the versatility of non-polar solvents in preserving fluorescence memory activity (figure 9).

The successful demonstration of long-term stability and PL retention shows how effective our encapsulation approach is in preventing material degradation. This is important for the continued development of durable and efficient perovskite-based technologies. By addressing issues like moisture, heat and oxygen exposure, our method improves the stability of perovskite materials, which is critical for their use in applications such as solar cells and light-emitting devices.

This work has clear implications for the commercial viability of perovskite technologies. By improving the durability of these materials, we help make them more reliable and cost-effective for long-term use, which could make perovskite-based devices more competitive in the market. Additionally, enhancing material stability can reduce the frequency of replacements, potentially lowering costs and minimizing waste. This has environmental benefits as well since fewer materials would need to be disposed of over time.

Our study also provides important insights into how to improve the longevity and performance of perovskite materials, which are essential for their practical application in optoelectronic devices. Future research will help refine our encapsulation strategies and explore alternative methods that could further enhance the stability and performance of these materials. By experimenting with different encapsulation techniques and solvent combinations, we can improve the overall durability and reliability of perovskite-based technologies in real-world conditions.

We believe this study not only contributes to improving the stability of perovskite materials but also brings us closer to overcoming the challenges that have hindered their widespread use. With further research, these materials could become more reliable, cost-effective and environmentally friendly, supporting their adoption in a range of commercial and industrial applications.

## 5. Conclusion

The synthesis and characterization of high molecular weight PTBr-encapsulated FAPbBr_₃_ (Pv) composites represent a significant advancement in the field of optoelectronic materials. The incorporation of PTBr into the FAPbBr_₃_ composite structure offers several notable advantages, including enhanced environmental stability and reduced quenching effects related to optoelectronic behaviour. The encapsulation of FAPbBr_₃_ creates a protective environment that shields the perovskite nanocrystals from environmental factors, particularly moisture, which is known to degrade performance. This enhanced stability is critical for ensuring the long-term reliability—over 1 year—and commercial viability of devices based on these composites, such as solar cells and LEDs.

Moreover, the conjugated nature of PTBr contributes to improved charge transport properties within the composite by enhancing electrical conductivity. This aspect is particularly significant for optoelectronic devices, where efficient charge transport is essential for optimal performance. The synergistic combination of FAPbBr_₃_ (Pv) and PTBr composites holds great promise for overcoming challenges associated with traditional FAPbBr_₃_ and similar perovskite materials, making them more suitable for practical applications in optoelectronics.

The development of these composite materials opens new avenues for designing and fabricating robust, high-performance devices with enhanced stability and functionality. Additionally, FAPbBr_₃_–PTBr composites are well-suited for implementation in wearable technology, flexible electronics and sensor-based applications.

Looking ahead, future research should explore various strategies to maximize and broaden the applicability of this encapsulation approach. Investigating lead-free perovskite materials encapsulated with conducting polymers could contribute to environmentally conscious advancements in the field. Furthermore, studies focusing on the scalability of this method for large-scale production will be crucial for commercial development. By emphasizing the dual benefits of thermal and environmental stability provided by conducting polymers, this research has the potential to influence future directions in the design and optimization of perovskite-based optoelectronic devices.

## Data Availability

The authors declare that the data supporting the findings of this study are available within the article and its electronic supplementary material [39]. Should any raw data files be needed in another format they are available from the corresponding author upon reasonable request.
